# Ultra-processed food consumption during pregnancy and emotional and behavioral problems in offspring

**DOI:** 10.1007/s00787-026-03044-0

**Published:** 2026-04-29

**Authors:** Nerea Becerra-Tomás, Josefa Canals Sans, Cristina Jardí, Núria Voltas, Carmen Hernández-Martínez, Victoria Arija

**Affiliations:** 1 Departament de Ciències Mèdiques Bàsiques, Unitat de Salut Pública i Epidemiologia Nutricional, Nutrition and Mental Health (NUTRISAM) research group, Universitat Rovira i Virgili, Carrer St. Llorenç, 21, Reus, 43201 Spain; 2 Institut de Recerca Biomèdica Catalunya Sud (IRBCatSud), Reus, Spain; 3 Department of Psychology, Nutrition and Mental Health (NUTRISAM) research group, Universitat Rovira i Virgili, Tarragona, Spain; 4 Department of Psychology, Research Center for Behavioral Assessment (CRAMC), Universitat Rovira i Virgili, Tarragona, Spain; 5 Collaborative Group on Lifestyles, Nutrition, and Tobacco (CENIT), Institut d’Investigació en Atenció Primària IDIAP Jordi Gol, Institut Català de la Salut (ICS), Reus, Spain

**Keywords:** Ultra-processed food, Pregnancy, Child development, Behavioral problems

## Abstract

Evidence on prenatal ultra-processed food (UPF) consumption and offspring psychopathological problems remains limited. Therefore, this study assessed the associations between UPF consumption during pregnancy and offspring emotional and behavioral problems. The potential impact of theoretically replacing UPFs with unprocessed or minimally processed foods was also examined using simulation models. The analyses included 201 mother-child pairs from the ECLIPSES study. Dietary intake was assessed using a validated food frequency questionnaire at 12, 24, and 36 weeks of gestation. UPF consumption (%grams/day) was estimated using the NOVA classification system. Children’s behavior was assessed at age 4 using the parent-reported Child Behaviour Checklist (1.5 to 5 years), with outcomes defined as borderline/clinical scores. Multivariable-adjusted logistic regression models were fitted. Every 10% grams/day increase in prenatal UPF consumption was associated with higher odds of having children with borderline/clinical scores on several syndrome and broad-band scales: emotionally reactive (Odd ratios [OR]: 1.67; 95% Confidence interval [CI]: 1.06–2.63), aggressive behavior (OR 2.13; 95% CI: 1.18–3.84), internalizing problems (OR: 1.56 95%CI: 1.06–2.31), externalizing problems (OR: 2.00; 95% CI: 1.31–3.07) and total problems (OR: 1.68; 95%CI: 1.12–2.51). Associations were also observed with DSM-oriented scales, including depressive (OR: 2.08; 95%CI: 1.25–3.45), attention deficit/hyperactivity (OR: 1.71; 95%CI: 1.08–2.68) and oppositional defiant problems (OR: 1.85; 95%CI: 1.09–3.13). Replacing 10%grams/day of UPF with equal amounts of unprocessed or minimally processed foods was associated with lower likelihood of children presenting the aforementioned borderline/clinical scores. The findings suggest that prenatal UPF consumption might be associated with adverse emotional and behavioral outcomes in the offspring.

**Supplementary Information:**

The online version contains supplementary material available at https://doi.org/10.1007/s00787-026-03044-0.

## Introduction

Ultra-processed foods (UPFs), according to the NOVA classification system, are considered industrially formulated products composed mainly of food substances (either derived or extracted from food) and additives, with little or no amount of unprocessed or minimally processed foods [1, 2]. These food products are designed to be ready-to-eat and highly appealing and are likely to displace other food groups from the diet. It is noteworthy that, due to their formulation, UPFs are of low nutritional quality, as they are high in energy, sodium, unhealthy fats, and added and free sugars, while low in some micronutrients, fiber and protein [3, 4]. Moreover, UPFs contain other substances (colorants, emulsifiers, artificial sweeteners, and flavors among others) [5] with potential adverse health effects [6–11].

In recent years, there has been increasing evidence linking UPF consumption to the development of different diseases [12–16]. In this context, a recent umbrella review found strong meta-analytical evidence supporting an adverse association between UPF consumption and all-cause and cardiovascular disease mortality, mental disorders, overweight/obesity, and type 2 diabetes mainly in adults [17]. However, the potential impact of UPF consumption at critical periods of high vulnerability, such as in early life, particularly during gestation, is still poorly investigated [18].

The in-utero phase is a sensitive period where maternal nutrition plays an important role in fetal growth and development, including brain development, with potential lifelong consequences [19]. In this context, the impact of maternal diet on cognitive and behavioral outcomes of offspring has been of special interest [20, 21]. A growing body of literature has examined overall maternal diet quality during pregnancy, showing that unhealthy dietary patterns may be associated with adverse emotional and behavioral outcomes in offspring [22–27]. These studies provide important and valuable insights into the role of maternal nutrition in early neurodevelopment. However, most previous research has not specifically examined the maternal diet according to the level of food processing, a factor that is also important to consider. In this context, given that UPFs are characterized by a high level of processing and low nutritional quality, it is important to understand the impact of their consumption during pregnancy on offspring emotional and behavioral development, as such problems, when manifested in early childhood, can be precursors of mental health issues throughout life [6, 28]. Despite this, studies on this subject are scarce [29]. To the best of our knowledge, only one previous prospective study has analyzed the association between total UPF consumption during pregnancy and offspring attention deficit hyperactivity disorder (ADHD) symptoms at 8 years of age [30], showing a negative impact of UPFs consumption around mid-gestation on child ADHD symptom scores. However, no previous research has assessed the impact on other symptom scales related to behavioral development beyond ADHD. In addition, the effect of substituting UPFs with other minimally processed or unprocessed foods in epidemiologic studies has not been evaluated previously. Therefore, the main aim of the present study was to assess the association between UPF consumption during pregnancy, evaluated by the NOVA classification system, and a broad spectrum of psychopathological problems, encompassing both emotional and behavioral problems, in 4-year-old children. We also analyzed the theoretical impact of substituting UPFs with an equivalent proportion of unprocessed or minimally processed foods in a simulation model.

## Materials and methods

### Study population

The present study included data from the ECLIPSES study (Ensayo CLInico Para Suplementar con hierro a EmbarazadaS), a previously published community randomized controlled trial, which was analyzed as if it were an observational prospective cohort. Briefly, the ECLIPSES study was conducted in Tarragona (Spain) between 2013 and 2017, with the main aim of determining the best level of efficacy of iron supplementation adapted to hemoglobin levels in early pregnancy that would be optimal for the health of the mother-child dyad [31]. In total, 791 healthy pregnant women over 18 years of age and less than 12 weeks of gestation were recruited from 12 primary care centers of the Catalunya Sexual and reproductive health care centers (ASSIR) of the Catalan Institute of Health. Additional information on the study protocol, as well as the inclusion and exclusion criteria, is available elsewhere [31]. A total of 319 pregnant women and their offspring participated in the subsequent phase of the study at the 4-years follow-up. For the present analysis, we included a subsample of 201 pregnant women and their children with available information on dietary intake during pregnancy and behavioral assessment of the child at age of 4 years. The study was designed in agreement with the Declaration of Helsinki and the Tokyo update. The protocol was approved by the Ethical Committee of the Jordi Gol Institute for Primary Care Research and the Pere Virgili Institute for Health Research. Written informed consent was obtained from all women included in the study.

### Prenatal UPFs assessment

Maternal dietary intake was assessed at 12, 24, and 36 weeks of gestation using a self-administered 45-item food frequency questionnaire (FFQ) [32] previously validated in our population. This questionnaire yielded data on frequency consumption in times per month or week, which was converted into grams per day using the average consumption ration observed in our population. The energy and nutrient consumption was calculated using the REGAL (Répertoire Général des Aliments) food composition Table [33], complemented by the Mataix Verdú Spanish food composition Table [34].

We classified foods and beverages using the NOVA classification system, which comprises four groups based on their degree of processing [2, 35]. Two independent research dietitians (NB-T and CJ) assigned the 45 food and beverage items included in the FFQ to the corresponding NOVA groups: Group 1, comprising unprocessed or minimally processed foods (e.g., vegetables, fruits, milk, potatoes, and rice); Group 2, comprising processed culinary ingredients (no items were assigned to this group, as these were not captured by the FFQ); Group 3, comprising processed foods (e.g., cheese, canned fruits, and bread); and Group 4, comprising UPFs (e.g., sugar-sweetened beverages, ice creams, cold meats, and pastries). The complete classification of FFQ items is provided in Supplementary Table 1. Any discrepancies were resolved through consensus. For the present study, we focused mainly on the UPF category. To estimate the total UPF consumption, we summed the amount (in grams per day) of each food item included in group 4 of the NOVA classification system and expressed it as the relative contribution to the total daily food intake in grams (%grams/day). This approach was chosen to also capture the non-nutritive factors of the UPFs (e.g., additives, contaminants, etc.). Daily UPF consumption was estimated for each trimester, and for the present analyses, the consumption during the whole gestational period was calculated as the average of the three trimesters.

### Child emotional and behavioral assessment

Children emotional and behavioral problems at 4 years was assessed by means of the Child Behaviour Checklist for ages 1.5 to 5 years (CBCL 1½−5) [36]. This parent-reported psychopathology test includes 99 items with 3 possible answers (never or hardly ever; sometimes true, very often true) and one open-ended item to report problems not included in the form. These items are grouped into seven syndrome scales (emotionally reactive, anxious/depressed, somatic complaints, withdrawn, sleep problems, attention problems and aggressive behavior); three broad-band scales (externalizing, internalizing, and total problems); and five DSM5 (Diagnostic and Statistical Manual of Mental Disorders)-oriented scales (depressive problems, anxiety problems, autism spectrum problems, attention-deficit/hyperactivity problems and oppositional defiant problems). For the present study, we included all syndrome scales but sleep problems, as they are not part of internalizing, externalizing, or total problems. T-scores were calculated for every scale in accordance with the scores of the Spanish population. For syndrome and DSM-oriented scales, T-scores 65–69 were considered borderline, while scores of 70 or higher were within the clinical range. For broad-band scales, scores of 60–64 were classified as borderline, whereas T-scores 65 or higher were considered within the clinical range. The Spanish validation of the CBCL1.5-5 has shown adequate internal consistency and good discriminative ability in the detection of disruptive and internalizing disorders in preschoolers [37]. For the present analyses, the outcome is defined as an alteration in the CBCL1.5-5 scales, based on having a score within the borderline or clinical ranges.

### Other covariables assessment

Midwives and nutritionists collected maternal data during personal interviews and using specific questionnaires. At the first trimester, information on age and educational level (primary, secondary, or university studies) was retrieved. Moreover, at each trimester visit (mean gestational ages: 11.9 ± 0.69, 19.9 ± 0.85, and 33.0 ± 0.99 weeks for the first, second and third trimesters, respectively), the following maternal data were gathered: (a) height and weight (which were used to calculate body mass index [BMI]; classified according to the current WHO BMI cut-offs (< 25 kg/m^2^, ≥ 25 to < 30 kg/m^2^ and ≥ 30 kg/m^2^); (b) physical activity which was assessed using the short version of the International Physical Activity Questionnaire (IPAQ-S) [38]; (c) smoking status; (d) maternal anxiety score during pregnancy which was evaluated by means of the State-Trait Anxiety Inventory (STAI) [39], which measures two distinct concepts, “state” and “trait”, and we focused our analyses on the state anxiety scores; and (e) dietary intake, which was assessed through the FFQ [32], including total kcal intake, saturated fatty acids, free sugar, and sodium intake. Furthermore, the relative Mediterranean Diet (rMedDiet) adherence score during pregnancy was also estimated, following the methodology previously described elsewhere [40].

Information on mode of delivery (vaginal or cesarean) was also collected.

Finally, offspring variables included sex (girls, boys), gestational age in weeks (at each trimester and at birth), and breastfeeding duration in months.

### Statistical analyses

Baseline characteristics of the study population were expressed as mean ± standard deviation (SD) or frequency and percentages for quantitative and categorical variables, respectively.

Logistic regression analyses were fitted to explore the association (Odd ratios [OR] and 95% Confidence Intervals [CI]) between UPFs consumption modeled as continuous (per 10%grams/day increment) during pregnancy and childhood emotional and behavioral problems. We performed crude models and multivariable models adjusted for early pregnancy BMI (<25, ≥25 to <30, ≥30 kg/m^2^), maternal age (in years), maternal smoking during pregnancy (smoker, non-smoker), maternal education (primary studies, secondary studies, university studies), intervention group (20 mg/d, 40 mg/d or 80 mg/d of iron), maternal anxiety score during pregnancy (in continuous), child’s sex (girl or boy), child's age at CBCL evaluation (in years) and delivery mode (cesarean or vaginal). Covariates were selected based on a previous literature review and using a causal directed acyclic graph (DAG) approach [41] (Supplementary Fig. 1).

As a secondary analysis, we examined UPF consumption separately for the first, second, and third trimesters of pregnancy. We fitted multivariable logistic regression models for each trimester using the same set of covariates as in the main analyses.

We performed a sensitivity analysis to assess the association between UPF consumption during pregnancy and emotional and behavioral problems in offspring, independent of diet quality, energy content and nutrient profile by alternately including in the multivariable adjusted model the following covariables: (1) adherence to the Mediterranean diet and energy intake; (2) free sugar intake; (3) sodium intake; (4) saturated fatty acid intake.

We additionally conducted a multivariable analysis to examine, using a theoretical substation model, the estimated impact of replacing 10%grams/day of UPFs with 10%grams/day of unprocessed or minimally processed food during pregnancy on psychological problems of the offspring. For this purpose, both variables were included simultaneously as continuous variables into the same model. The difference in their beta-coefficients, variance, and covariance was used to calculate the OR and 95% CIs for the substitution association.

In the present analysis, data were missing for the covariable State-Trait maternal anxiety score during pregnancy (4,9%). As the proportion of missingness was low, we imputed missing data to the mean value [42].

All statistical analyses were performed using STATA statistical software version 15.0 (StataCorp LP, Tx. USA). Significant level was set at a two-sided p-value of less than 0.05.

## Results

The general characteristics of the study population are depicted in Table 1. Briefly, the pregnant women had a mean age of 32 ± 4 years, with 58% having a BMI < 25 kg/m². A total of 46% of participants had completed university studies. The mean UPF consumption during pregnancy was 19.67%g/day, with sugar-sweetened beverages being the primary contributors to total UPF intake followed by sugary products. Regarding the offspring, 49% were boys, with a mean age of 4 ± 0.3 years old. The mean gestational age at birth was 39 ± 2 weeks, and the mean breastfeeding duration was 11 ± 13 months.

**Table 1:** Baseline characteristics of the study population (*n* = 201)

General characteristics of the mother		
Age (years)	32 ± 4	
Early pregnancy BMI (kg/m^2^), n (%)^¥^		
Normal weight	116 (57.7)	
Overweight	57 (28.4)	
Obesity	28 (13.9)	
Educational level, n (%)		
Primary or less	36 (17.9)	
Secondary	72 (35.8)	
University	93 (46.3)	
Smoke during pregnancy (yes), n (%)	33 (16.4)	
Anxiety score during pregnancy (STAI score)	14.05 ± 8.14	
Delivery mode, n (%)		
Vaginal	163 (81.1)	
Cesarean	38 (18.9)	
Ultra-processed food intake during pregnancy (%g/day)		
Total ultra-processed food intake	19.67 ± 8.62	
Breakfast cereals	0.83 ± 0.94	
Sugary products	3.19 ± 1.97	
Dairy-based products	2.89 ± 2.45	
Processed meat	2.48 ± 1.30	
Sugar-sweetened beverages	6.98 ± 6.20	
Artificially Sweetened Beverages	1.82 ± 3.38	
Alcoholic drinks	0	
Pre-cooked food	1.02 ± 0.72	
Savory snacks	0.46 ± 0.37	
Characteristics of the child		
Age (years)	4 ± 0.3	
Sex (boy), n (%)	99 (49)	
Gestational age at birth (weeks)	39 ± 2	
Breastfeeding (months)	11 ± 13	
Child behavioral assessment (CBCL 1.5 T-score)		
	mean ± SD	Children with T-scores within the borderline or clinical range, n (%)
Emotionally reactive	56.21 ± 9.04	38 (18.9)
Anxious/depressed	56.22 ± 7.52	30 (14.9)
Somatic complaints	55.49 ± 6.91	30 (14.9)
Withdrawn	58.22 ± 7.80	38 (18.9)
Attention problems	58.87 ± 7.26	51 (25.4)
Aggressive behavior	55.63 ± 7.45	22 (10.9)
Internalizing problems	55.10 ± 12.02	76 (37.8)
Externalizing problems	53.64 ± 11.08	61 (30.3)
Total problems	54.81 ± 12.29	66 (32.8)
DSM-Depressive problems	56.75 ± 7.38	35 (17.7)
DSM-Anxiety problems	57.56 ± 8.29	38 (18.9)
DSM-Autism spectrum problems	57.68 ± 7.53	45 (22.4)
DSM-Attention deficit/Hyperactivity problems	57.44 ± 7.87	41 (20.4)
DSM-Oppositional defiant problems	54.98 ± 6.87	26 (12.9)

Values are expressed in mean ± standard deviation or frequency (percentages), as appropriate

^¥^ Measurements were taken at a mean gestational age of 11.9 ± 0.69 weeks

BMI: Body Mass Index; CBCL 1.5-5: Child Behavior Checklist 1.5-5; DSM: Diagnostic and Statistical Manual of Mental Disorders

The associations between UPF consumption during pregnancy and children’s emotional and behavioral problems are presented in Table 2. Regarding syndrome scales, in the fully adjusted model, each 10%grams/day increase in prenatal UPF consumption was associated with 66% higher odds of children exhibiting borderline/clinical scores for emotionally reactive (OR_10%g/day_: 1.67; 95%CI: 1.06, 2.63; *p* = 0.03) and 2.13 higher odds of aggressive behavior (OR_10%g/day_: 2.13; 95%CI: 1.18, 3.84; *p* = 0.01). UPF consumption during pregnancy was not associated with the other syndrome scales (anxious/depressed, withdrawn, and attention problems). For the broad-band scales, the ORs for having children with borderline/clinical scores for internalizing, externalizing and total problems for each 10%grams/day increase in prenatal UPF consumption were 1.56 (95%CI: 1.06, 2.31; *p* = 0.03), 2.00 (1.31, 3.07; *p* = 0.01), and 1.68 (1.12, 2.51; *p* = 0.01), respectively. In terms of DSM-oriented scales, each 10%grams/day increase in prenatal UPF consumption was associated with two-fold higher likelihood of having children with depressive problems (OR_10%g/day_: 2.08; 95%CI: 1.25, 3.45; *p* < 0.01), a 1.71-fold higher likelihood of having children with attention deficit/hyperactivity problems (OR_10%g/day_: 1.71; 95%CI: 1.08, 2.68; *p* = 0.02), and a 1.85-fold higher likelihood of having children with oppositional defiant problems (OR_10%g/day_: 1.85; 95%CI: 1.09, 3.13; *p* = 0.02). There were no associations between UPF consumption during pregnancy and the other DSM-oriented scales.

**Table 2:** Multivariable-adjusted odd ratios and 95% confidence interval for the association between prenatal ultra-processed food consumption (10% grams/day increment) during pregnancy and emotional and behavioral problems of the offspring at 4 years (*n* = 201).

	Unadjusted model		Fully adjusted model*	
	OR (95%CI)	*P*-value	OR (95%CI)	*P*-value
Syndrome Scales				
Emotionally reactive	1.60 (1.08, 2.38)	0.02	1.67 (1.06, 2.63)	0.03
Anxious/depressed	1.20 (0.77, 1.84)	0.41	1.22 (0.74, 2.03)	0.44
Somatic complaints	1.52 (0.99, 2.34)	0.05	1.55 (0.94, 2.55)	0.09
Withdrawn	1.37 (0.92, 2.03)	0.12	1.25 (0.79, 1.99)	0.35
Attention problems	1.40 (0.97, 2.01)	0.07	1.35 (0.90, 2.03)	0.15
Aggressive behavior	1.96 (1.21, 3.16)	0.01	2.13 (1.18, 3.84)	0.01
Broad-band scales				
Internalizing problems	1.57 (1.11, 2.21)	0.01	1.56 (1.06, 2.31)	0.03
Externalizing problems	1.82 (1.27, 2.62)	< 0.01	2.00 (1.31, 3.07)	0.01
Total problems	1.53 (1.08, 2.17)	0.02	1.68 (1.12, 2.51)	0.01
DSM-Oriented Scales				
Depressive problems	1.86 (1.23, 2.82)	< 0.01	2.08 (1.25, 3.45)	< 0.01
Anxiety problems	1.19 (0.80, 1.77)	0.40	1.13 (0.72, 1.78)	0.60
Autism spectrum problems	1.38 (0.95, 2.01)	0.09	1.27 (0.81, 1.99)	0.29
Attention deficit/hyperactivity problems	1.68 (1.14, 2.49)	0.01	1.71 (1.08, 2.68)	0.02
Oppositional defiant problems	1.80 (1.15, 2.82)	0.01	1.85 (1.09, 3.13)	0.02

*Models adjusted for maternal early Body Mass Index (< 25, 25 to < 30, ≥30 kg/m2), maternal age (in years), maternal smoking during pregnancy (smoker, non-smoker), maternal education (primary studies, secondary studies, university studies), intervention group (20 mg/d, 40 mg/d or 80 mg/d), maternal anxiety score during pregnancy, child’s sex (girl, boy), child’s age at CBCL evaluation (in years) and delivery mode (cesarean or vaginal)

Abbreviations: CI, confidence interval; OR, Odd Ratio; UPF, Ultra-processed food

In analyses stratified by trimester of pregnancy, associations were observed in the same direction across trimesters, although estimates for the third trimester did not reach statistical significance (Supplementary Table 2).

The results remained similar after accounting for diet quality and energy intake, or nutrient content (sodium or saturated fatty acid intake). However, the associations with emotionally reactive scale, internalizing and total problems, as well as DSM-oriented depressive and attention deficit/hyperactivity problems became non-statistically significant after adjusting for the prenatal percentage of free sugar intake (Supplementary Table 3: model 3).

**Fig. 1:**
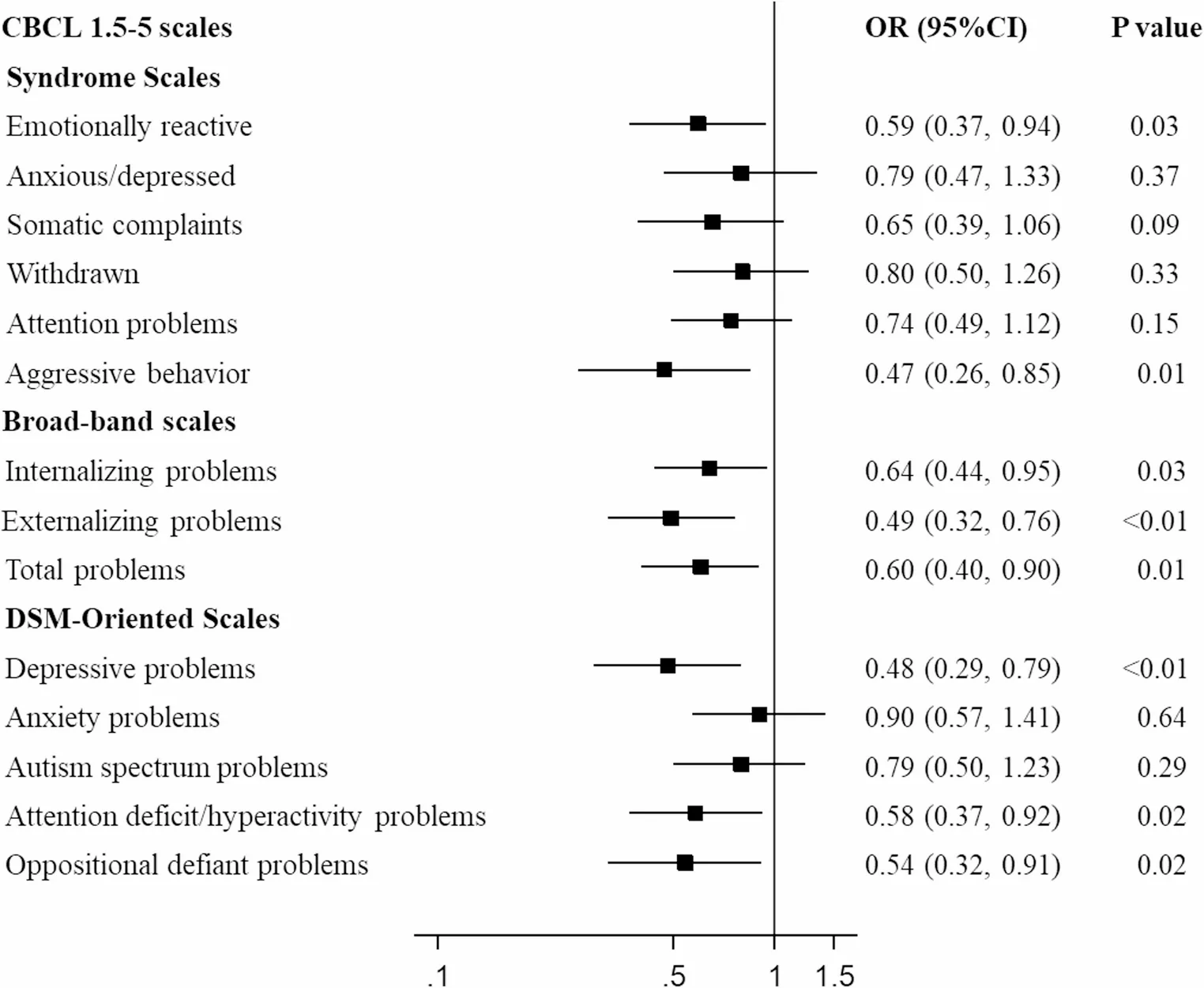
The impact of theoretical substitution of 10% grams/day of ultra-processed food with 10% grams/day of unprocessed or minimally processed food during pregnancy on emotional and behavioral problems of the offspring at 4 years. All logistic regression models were adjusted for maternal early pregnancy Body Mass Index (< 25, 25 to < 30, ≥30 kg/m^2^), maternal age (in years), maternal smoking during pregnancy (smoker, non-smoker), maternal education (primary studies, secondary studies, university studies), intervention group (20 mg/d, 40 mg/d or 80 mg/d), maternal anxiety score during pregnancy, child’s sex (girl, boy), child’s age at CBCL evaluation (in years) and delivery mode (cesarean or vaginal). Abbreviations: CBCL 1.5-5, Child Behavior Checklist 1.5-5; CI, Confidence Interval; OR, Odd Ratio

In theoretical substitution models, all significant adverse associations observed between UPF consumption during pregnancy and offspring and behavioural and emotional problems were significantly alleviated when replacing 10%grams/day of UPFs with an equal amount of unprocessed or minimally processed food during pregnancy (Fig. 1).

## Discussion

In the present analysis, an increased UPF consumption during pregnancy was associated with higher odds of behavioral and emotional problems in the offspring at the 4 years of age. Specifically, its consumption was adversely associated with borderline/clinical scores on the emotionally reactive and aggressive behavior syndrome scales; the broad band scales of internalizing, externalizing and total problems; and with the DSM-oriented scales of depressive, attention deficit/hyperactivity and oppositional defiant problems. A similar pattern was observed in secondary analyses using trimester-specific exposure in the first and second trimesters. Of note, based on the theoretical substitution models, replacing 10%grams/day of UPF for the same amount of unprocessed or minimally processed foods was associated with lower likelihood of having children with the aforementioned borderline/clinical scores. These findings have relevant implications, as these behaviors are early markers of future mental health problems [43]. In addition, identifying modifiable risk factors in early life (including nutritional factors) is essential, as this knowledge may offer valuable opportunities for the primary prevention of these emotional and behavioral problems.

To our knowledge, the present study is the first evaluating the association between total prenatal UPF consumption and a broad spectrum of psychopathological problems, encompassing both emotional and behavioral problems, in children beyond ADHD-related symptoms. Previous research has mainly focused on assessing total maternal diet quality, showing that unhealthy dietary patterns (emulating a Westernized diet) might be associated with subsequent risk for emotional and behavioral problems in the offspring [22, 24, 25]. However, even though UPFs are a hallmark of these dietary patterns, these previous studies have not distinguished the level of industrial food processing. So far, only one previous study has assessed the relationship between maternal total UPF consumption during pregnancy and behavioral outcomes, focusing on ADHD symptoms, in the offspring at 8 years of age [30]. Their results are in line with our findings, as a high UPF consumption (expressed as a percentage of total energy intake) was also associated with increased ADHD symptom sores in their children. Interestingly, a previous study within the Avon Longitudinal Study of Children and Parents (ALSPAC), showed that consuming at least one serving per day of salty processed foods (not including sugary processed foods) during pregnancy and consumption of these foods by the child at 3 years of age was associated with increased hyperactivity between ages 4 and 10 [44]. These results are in line to some extent with those found in our study, despite the fact that we assessed total prenatal UPF consumption rather than solely savory processed foods during pregnancy and at 3 years of age, as we also observed increased odds of children having borderline/clinical scores on ADHD problems per each 10%grams/day increment in UPF consumption.

In the present study, trimester-specific analyses were consistent with the main analysis based on average UPF consumption during pregnancy, but suggested a slightly differentiated pattern of associations. Statistically significant associations were mainly observed for UPF consumption during the first and second trimesters, while associations for the third trimester were attenuated and no longer statistically significant. This pattern may suggest the existence of sensitive windows during early and mid-gestation, when key neurodevelopmental processes take place [45, 46]. Moreover, nutritional exposures during these stages may have a stronger programming effect on infant development than exposures occurring later in pregnancy [47].

Notably, for the first time using theoretical substitution models, we have shown that an estimated substitution of UPF with minimally or unprocessed food is associated with lower probability of having children with psychopathological problems. Our findings, together with those from previous studies, underscore the importance of providing specific dietary guidance during antenatal care aimed at reducing UPF consumption to mitigate the risk of emotional and behavioral problems in offspring, rather than relying solely on broad recommendations to adopt a less Westernized dietary pattern.

The mechanisms underlying these adverse associations have not yet been fully elucidated, although some hypotheses exist. One is the negative impact of UPF consumption on diet quality, due to their poor nutritional quality and the displacement of other healthy choices. Increased UPF consumption has been linked to lower consumption of protein, fiber, and essential micronutrients such as zinc, magnesium, and vitamins A, D, E, C, B12, and niacin [48]. These compounds are crucial for fetal neurological development, as an adequate supply during pregnancy is needed for the brain to form and mature properly [49]. UPF consumption has also been related to higher intakes of sodium, saturated fats, and added sugars, which have been suggested as potential contributors to these associations. Excessive maternal consumption of these nutrients might negatively influence the structure and function of the developing brain, which could lead to later behavioral problems in children [21]. Nonetheless, in our study, the adjustment for most of these dietary factors (including overall diet quality, saturated fat and sodium) did not explain away the associations between UPF and behavioral outcomes. However, some associations, mainly with internalizing problems, were no longer significant after adjustment for prenatal free sugar intake, suggesting that this nutrient, a key component of UPFs, may partly contribute to these associations. Since free sugar is a major component of UPFs, it is difficult to disentangle the independent effect of sugar from the overall impact of UPF consumption. Further mechanistic studies specifically designed to evaluate these outcomes are warranted.

Beyond the poor nutritional quality, growing evidence also indicates that various factors introduced during food processing may adversely affect children’s conduct. For instance, UPFs contain additives (e.g., nonnutritive sweeteners, emulsifiers, preservatives), which might disrupt gut microbiome and activate inflammatory pathways [50]. Elevated maternal inflammatory status has been shown to negatively impact fetal brain development and neurobehavioral outcomes during childhood [51–53]. Furthermore, UPFs might contain contaminants, such as phthalates and bisphenols, which can migrate from food packaging materials [54]. Prenatal exposure to these compounds has been associated with adverse behavioral and executive functioning outcomes in children [55].

Our study has limitations that should be acknowledged. Firstly, we used dietary data obtained from an FFQ to classify foods according to their degree of processing based on the NOVA classification system. Since FFQs are not specifically designed to capture UPF consumption, there is a possibility that actual UPF consumption may be miscalculated. In our case, the FFQ did not allow differentiation between homemade and ready-made vegetable soups, nor between processed and unprocessed minced meat or hamburgers, and the chocolate item included different types of products, which may have introduced some degree of misclassification. Secondly, although we adjusted for multiple potential confounders in our statistical analyses, the possibility of residual confounding cannot be entirely ruled out. Thirdly, the study population consisted of Spanish mother–child pairs from a Mediterranean region, which may limit the generalizability of our findings to other populations with different sociodemographic characteristics. Fourthly, although we used a validated instrument to assess children’s behavior, the evaluation was based exclusively on parent-reported data instead of clinical evaluations. Fifth, pre-pregnancy BMI data were not available. Maternal BMI was assessed during the first trimester (around gestational week 12). Although these measures are correlated, some degree of misclassification cannot be ruled out. Finally, due to the observational design of the study, causality cannot be established. However, our study also presents some strengths, such us the use of repeated dietary measurements across all three trimesters of pregnancy, which allowed for the calculation of an average consumption and provided a more accurate representation of overall maternal diet, while also reducing intraindividual variation and measurement error. Other strengths include the control for multiple potential confounding factors in the analyses, the prospective study design, and the relatively large sample size, all of which contribute to the robustness of our findings.

## Conclusions

In conclusion, the present study provides new insights on the detrimental impact of prenatal UPF consumption on offspring emotional and behavioral problems and suggests that replacing UPF with minimally or unprocessed foods may reduce the likelihood of such outcomes. From a public health perspective, these findings highlight the importance of promoting the consumption of minimally processed foods, while reducing UPFs, in critical periods of high vulnerability, such as during gestation. In addition, the results may help inform nutritional guidelines for pregnant women and support the development of evidence-based interventions aimed at promoting optimal behavioral development in early childhood. Future research is needed to replicate these results in cohorts from other settings and to explore the underlying mechanisms of the observed associations beyond the nutritional profile of UPFs.

## Supplementary Information

Below is the link to the electronic supplementary material.

Supplementary Material 1

## Data Availability

The data that has been used is confidential. The datasets generated and/or analyzed in the current study are available from the corresponding author upon reasonable request.
