# Supplementary material for: Ultra-processed food consumption during pregnancy and emotional and behavioral problems in offspring
Source: Eur Child Adolesc Psychiatry. 2026 Apr 29;35(8):2633–43. doi: 10.1007/s00787-026-03044-0 (PMC13652858; doi:10.1007/s00787-026-03044-0)
Supplement: Supplementary file 1 [file 787_2026_3044_MOESM1_ESM.pdf]

Supplementary material

Title: Ultra-processed food consumption during pregnancy and emotional and behavioral problems in offspring.

Journal: European Child & Adolescent Psychiatry

Authors: Nerea Becerra-Tomás[1,2], Josefa Canals Sans[2,3,4], Cristina Jardí[1,2], Núria Voltas[2,3,4], Carmen Hernández-Martínez[2,3,4] and Victoria Arija[1,2,5]

Affiliations:

1. Universitat Rovira i Virgili, Departament de Ciències Mèdiques Bàsiques, Nutrition and Mental Health (NUTRISAM) research group; Unitat de Salut Pública i Epidemiologia Nutricional, Reus, Spain

2. Institut d'Investigació Sanitària Pere Virgili (IISPV), 43005 Tarragona, Spain

3. Universitat Rovira i Virgili, Department of Psychology, Nutrition and Mental Health (NUTRISAM) research group, Tarragona, Spain

4. Research Center for Behavioral Assessment (CRAMC); Department of Psychology, Rovira i Virgili University, Tarragona, Spain

5. Institut Català de la Salut (ICS), Collaborative Group on Lifestyles, Nutrition, and Tobacco (CENIT), Institut d´Investigació en Atenció Primària IDIAP Jordi Gol, Reus, Spain

Corresponding author:

Victoria Arija, Universitat Rovira i Virgili, Departament de Ciències Mèdiques Bàsiques, Nutrition and Mental Health (NUTRISAM) research group; Unitat de Salut Pública i Epidemiologia Nutricional, Reus, Spain, Carrer St. Llorenç, 21, 43201, Reus, Tarragona; email: victoria.arija@urv.cat

Content

Supplementary Table 1. Food classification according to the NOVA... 2

Supplementary table 2. Multivariable logistic regression analysis for the association between percentage of ultra-processed food intake (10% increment) at each trimester and emotional and behavioral problems of the offspring at 4 years 3

Supplementary table 3. Multivariable logistic regression analysis for the association between percentage of ultra-processed food intake (10% increment) and emotional and behavioral problems of the offspring at 4 years (n=201). Sensitivity analyses adjusted for nutritional factors.. 4

Supplementary Figure 1. Directed Acyclic Graph (DAG) model. 6

Supplementary Table 1. Food classification according to the NOVA

**Group 1: Unprocessed or minimally processed foods**	Milk; yogurt; lettuce, tomato or escarole; green beans, spinach, or chard; garnish vegetables; potatoes (baked, fried or boiled); legumes; rice; pasta; soup and vegetable soups; egg; poultry; red meat (lamb, beef, pork); white fish; fatty fish; seafood (mussels, prawns, squids…); citric fruits; other fruits (apple, pear, banana,); natural fruit juices; nuts
**Group 2: Culinary ingredients**	No items were assigned to this group, as these were not captured by the food frequency questionnaire
**Group 3: Processed foods**	Bread; fresh cheese; mature or semi-mature cheeses; canned fruits; wine; beer; alcohol-free beer
**Group 4: Ultra-processed food and drink products**	Chocolate; breakfast cereals; plain biscuits; chocolate biscuits; muffins and sponge cakes; pastries, doughnuts and croissants; processed meat (hamburger, minced meat, sausage, …); cold meat (processed meats such ham, chorizo, or similar); croquette, pizza or similar; commercial fruit juices; dairy desserts (custard, pudding or similar); cream or chocolate cakes; snacks; sweets; ice-creams; sugar-sweetened soft drinks; artificially- sweetened soft drinks; spirits (whisky, gin, cognac, …)

Supplementary table 2. Multivariable logistic regression analysis for the association between percentage of ultra-processed food intake (10% increment) at each trimester and emotional and behavioral problems of the offspring at 4 years

	First trimester		Second trimester		Third trimester	
**CBCL 1.5-5 scales**	**OR (95%CI)**	**P-value**	**OR (95%CI)**	**P-value**	**OR (95%CI)**	**P-value**
**Syndrome Scales**						
Emotionally reactive	1.30 (0.83, 2.05)	0.25	1.28 (0.84, 1.96)	0.25	1.04 (0.63, 1.71)	0.88
Anxious/depressed	0.98 (0.61, 1.60)	0.95	1.47 (0.90, 2.40)	0.12	0.86 (0.48, 1.56)	0.63
Somatic complaints	1.02 (0.63, 1.67)	0.93	1.45 (0.90, 2.34)	0.13	1.00 (0.56, 1,80)	0.99
Withdrawn	1.60 (0.99, 2.56)	0.05	1.66 (1.07, 2.59)	0.02	0.87 (0.54, 1.39)	0.56
Attention problems	1.16 (0.79, 1.72)	0.44	1.54 (1.04, 2.28)	0.03	1.17 (0.76, 1.79)	0.48
Aggressive behavior	1.70 (0.97, 3.01)	0.07	1.79 (1.03, 3.13)	0.04	1.07 (0.58, 1.98)	0.83
**Broad-band scales**						
Internalizing problems	1.28 (0.88, 1.87)	0.19	1.40 (0.98, 2.00)	0.07	1.28 (0.88, 1.88)	0.20
Externalizing problems	1.83 (1.21, 2.78)	<0.01	1.63 (1.10, 2.42)	0.02	1.48 (0.98, 2.23)	0.06
Total problems	1.58 (1.06, 2.36)	0.02	1.51 (1.03, 2.20)	0.03	1.19 (0.80, 1.78)	0.39
**DSM-Oriented Scales**						
Depressive problems	2.09 (1.24, 3.53)	0.01	2.72 (1.48, 5.00)	<0.01	1.28 (0.76, 2.15)	0.36
Anxiety problems	1.00 (0.64, 1.55)	0.99	1.14 (0.74, 1.76)	0.55	0.68 (0.39, 1.19)	0.18
Autism spectrum problems	1.52 (0.99, 2.35)	0.06	1.26 (0.84, 1.90)	0.26	0.82 (0.51, 1.32)	0.42
Attention deficit/hyperactivity problems	1.42 (0.92, 2.19)	0.11	1.90 (1.23, 2.93)	0.04	1.27 (0.81, 1.99)	0.30
Oppositional defiant problems	1.70 (1.00, 2.89)	0.04	1.44 (0.88, 2.37)	0.15	1.10 (0.64, 1.89)	0.74

Models adjusted for maternal early Body Mass Index (<25, 25 to <29, ≥30 kg/m2), maternal age (in years), maternal smoking during pregnancy (smoker, non-smoker), maternal education (primary studies, secondary studies, university studies), intervention group (20mg/d, 40mg/d or 80mg/d), maternal anxiety score at 1st, 2nd or 3rd trimester (as appropriate), child's sex (girl, boy), child's age at CBCL evaluation (in years) and delivery mode (cesarean or vaginal). Abbreviations: CI, confidence interval; OR, Odd Ratio; UPF, Ultra-processed food;

Supplementary table 3. Multivariable logistic regression analysis for the association between percentage of ultra-processed food intake (10% increment) and emotional and behavioral problems of the offspring at 4 years (n=201). Sensitivity analyses adjusted for nutritional factors.

	Model 1[a]		Model 2[b]		Model 3[c]		Model 4[d]	
**CBCL 1.5-5 scales**	**OR (95%CI)**	**P-value**	**OR (95%CI)**	**P-value**	**OR (95%CI)**	**P-value**	**OR (95%CI)**	**P-value**
**Syndrome Scales**								
Emotionally reactive	1.58 (0.99, 2.54)	0.06	1.68 (1.06, 2.67)	0.03	2.26 (0.82, 6.19)	0.11	1.68 (1.06, 2.67)	0.03
Anxious/depressed	1.18 (0.69, 1.99)	0.55	1.21 (0.72, 2.02)	0.47	0.53 (0.16, 1.72)	0.29	1.23 (0.74, 2.04)	0.43
Somatic complaints	1.37 (0.82, 2.29)	0.24	1.50 (0.90, 2.49)	0.12	1.87 (0.65, 5.43)	0.25	1.55 (0.93, 2.57)	0.09
Withdrawn	1.25 (0.77, 2.04)	0.37	1.29 (0.81, 2.07)	0.29	1.28 (0.46, 3.59)	0.63	1.26 (0.79, 2.01)	0.32
Attention problems	1.17 (0.76, 1.82)	0.47	1.37 (0.91, 2.07)	0.13	2.03 (0.82, 5.05)	0.13	1.35 (0.90, 2.03)	0.15
Aggressive behavior	2.40 (1.25, 4.57)	0.01	2.26 (1.23, 4.16)	0.01	3.52 (1.22, 10,17)	0.02	2.13 (1.18, 3.84)	0.01
**Broad-band scales**								
Internalizing problems	1.52 (1.02, 2.29)	0.04	1.57 (1.06, 2.33)	0.03	1.99 (0.85, 4.64)	0.11	1.56 (1.06, 2.31)	0.03
Externalizing problems	1.67 (1.06, 2.61)	0.03	2.00 (1.30, 3.07)	0.02	4.22 (1.60, 11.14)	<0.01	2.01 (1.31, 3.09)	0.01
Total problems	1.53 (1.00, 2.32)	0.05	1.68 (1.12, 2.52)	0.01	1.58 (0.66, 3.79)	0.30	1.68 (1.12, 2.51)	0.01
**DSM-Oriented Scales**								
Depressive problems	1.98 (1.17, 3.35)	0.01	2.21 (1.30, 3.73)	<0.01	2.76 (0.93, 8.22)	0.07	2.17 (1.30, 3.65)	<0.01
Anxiety problems	1.01 (0.63, 1.63)	0.96	1.10 (0.70, 1.75)	0.67	1.34 (0.50, 3.63)	0.56	1.14 (0.72, 1.80)	0.58
Autism spectrum problems	1.27 (0.79, 2.03)	0.32	1.33 (0.84, 2.09)	0.22	2.35 (0.87, 6.37)	0.09	1.28 (0.82, 2.00)	0.28
Attention deficit/hyperactivity problems	1.55 (0.96, 2.49)	0.07	1.74 (1.10, 2.75)	0.02	2.28 (0.83, 6.25)	0.11	1.71 (1.09, 2.69)	0.02
Oppositional defiant problems	1.83 (1.03, 3.21)	0.04	1.91 (1.12, 3.26)	0.02	2.68 (1.01, 7.06)	0.04	1.85 (1.10, 3.13)	0.02

a) Model 1 adjusted for maternal early Body Mass Index (<25, 25 to <29, ≥30 kg/m2), maternal age (in years), maternal smoking during pregnancy (smoker, non-smoker), maternal education (primary studies, secondary studies, university studies), intervention group (20mg/d, 40mg/d or 80mg/d), maternal anxiety score during pregnancy, child's sex (girl, boy), child's age at CBCL evaluation (in years) and delivery mode (cesarean or vaginal), Mediterranean Diet adherence (continuous score), total kcal intake (in tertiles).

b) Model 2 adjusted for maternal early Body Mass Index (<25, 25 to <29, ≥30 kg/m2), maternal age (in years), maternal smoking during pregnancy (smoker, non-smoker), maternal education (primary studies, secondary studies, university studies), intervention group (20mg/d, 40mg/d or 80mg/d), maternal anxiety score during pregnancy, child's sex (girl, boy), child's age at CBCL evaluation (in years) and delivery mode (cesarean or vaginal), saturated fatty acids (g/day).

c) Model 3 adjusted for maternal early Body Mass Index (<25, 25 to <29, ≥30 kg/m2), maternal age (in years), maternal smoking during pregnancy (smoker, non-smoker), maternal education (primary studies, secondary studies, university studies), intervention group (20mg/d, 40mg/d or 80mg/d), maternal anxiety score during pregnancy, child's sex (girl, boy), child's age at CBCL evaluation (in years) and delivery mode (cesarean or vaginal), free sugar intake (continuous as percentage of total energy intake).
d) Model 4 adjusted for maternal early Body Mass Index (<25, 25 to <29, ≥30 kg/m2), maternal age (in years), maternal smoking during pregnancy (smoker, non-smoker), maternal education (primary studies, secondary studies, university studies), intervention group (20mg/d, 40mg/d or 80mg/d), maternal anxiety score during pregnancy, child's sex (girl, boy), child's age at CBCL evaluation (in years) and delivery mode (cesarean or vaginal), sodium intake (mg/day). Abbreviations: CI, confidence interval; UPF, Ultra-processed food; β, beta-coefficient

Supplementary material

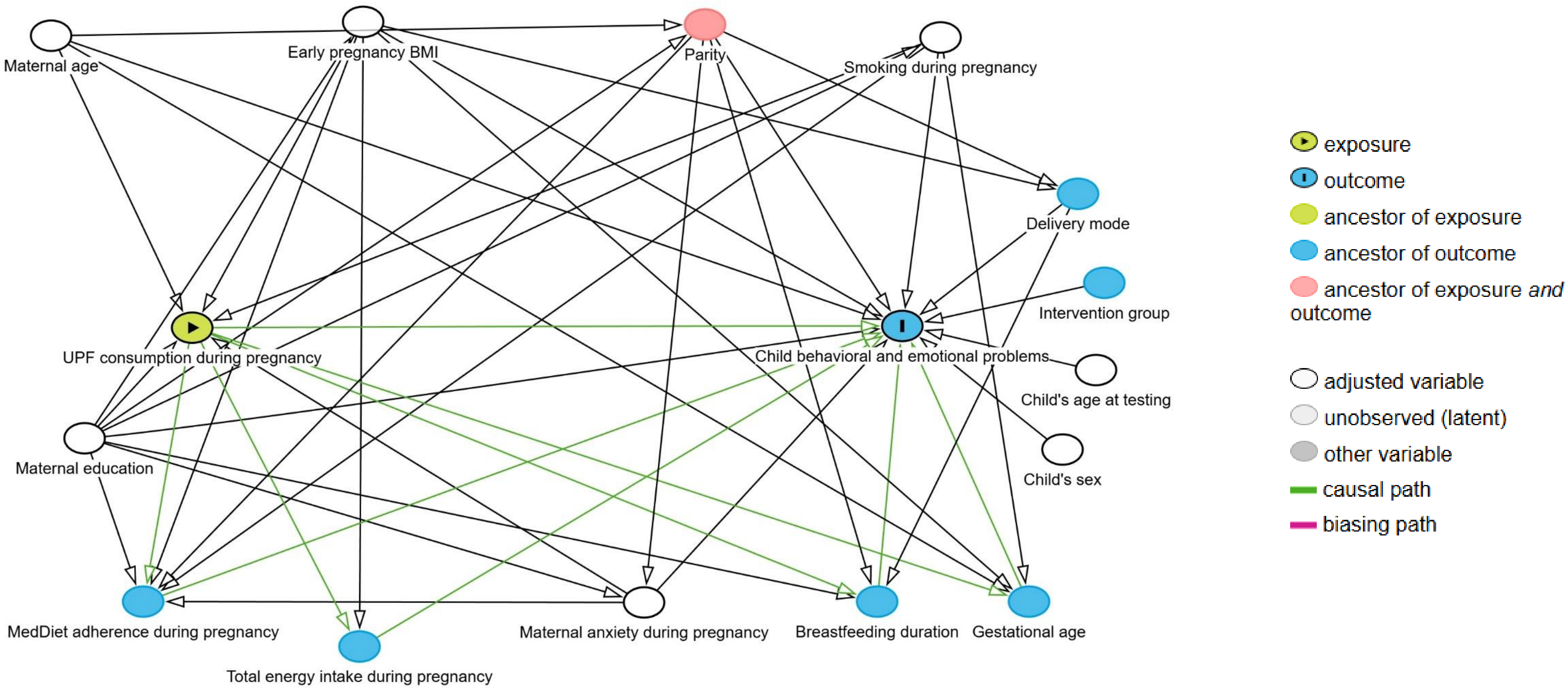

Supplementary Figure 1. Directed Acyclic Graph (DAG) model.
